# Gas burner experiments conducted in modern residential style structures

**DOI:** 10.1016/j.dib.2021.107624

**Published:** 2021-11-23

**Authors:** Mark McKinnon, Craig Weinschenk, Daniel Madrzykowski, Keith Stakes

**Affiliations:** UL Firefighter Safety Research Institute, Columbia, MD, US^1^

**Keywords:** Fire model CFD, Validation gas burner, Residential structures ventilation, Fire investigation

## Abstract

The fire modeling community currently lacks full-scale data from tests conducted in realistic residential-style structures. Controlled gas burner tests were conducted in purpose-built single- and two-story structures instrumented throughout with thermocouples, pressure transducers, and bi-directional probes. Experiments consisted of sequences of ventilation events. The data collected in these tests was intended to provide several new validation cases for the fire modeling community.

## Specifications Table

**Table:** 

Subject	Engineering
Specific subject area	Experimental Thermal and Fluid Sciences and Fire Safety Engineering
Type of data	Table
How data were acquired	Type K Thermocouples Bi-directional Probes Setra Model 264 Capacitive Differential Pressure Sensors
Data format	Raw
Parameters for data collection	13 gas burner experiments were conducted in a single-story (5) and a two-story (8) residential-style structure. Both structures were constructed in a large indoor laboratory with a volume of 18,920 m^3^ and a ventilation rate of 420 m^3^/min, which resulted in relatively quiescent initial conditions. Initial temperatures ranged from 18 °C to 25 °C for all experiments.
Description of data collection	The gas burner was supplied with a flow rate of natural gas to produce a 250 kW fire in the single-story experiments and a 500 kW fire in the two-story experiments. Thermocouple arrays with sensors ranging from the ceiling to the floor were used to measure temperatures. Arrays of bi-directional probes were used to measure gas flow velocities. Arrays of differential pressure sensors were used to measure static pressure relative to atmospheric pressure external to the structure. Experiments consisted of unique sequences of ventilation of exterior window and door openings.
Data source location	Experiments were conducted at the UL Large Fire Laboratory, Northbrook, IL, United States Latitude and longitude for collected data: 42.14616, −87.84650
Data accessibility	The raw data are archived in a github repository at. Data identification number: https://doi.org/10.5281/zenodo.5703080 Direct URL to the data: https://github.com/ulfsri/fsri-residential-gasburner-2016 The dataset citation is in Ref [1]
Related research article	M. McKinnon, C. Weinschenk. Validation of CFD Fire Model Pressure Predictions for Modern Residential Style Structures. Fire Safety Journal. 126 (2021) [2]

## Value of the Data

• These data provide a complete picture of fluid flow and gas-phase heat transfer in residential-style structures given a well-defined fire source and distinct sequences of ventilation events.
• These data may benefit fire model developers and practitioners, fire protection engineers, investigators, and firefighters.
• These data may be used to validate fire models and improve understanding of flow paths generated due to ventilation in residential structures.

## 1 Data Description

The github repository contains the data and supporting files from gas burner experiments conducted in single-story and two-story residential structures. The repository contains direc- tories for the raw data and event timestamps, supporting information for the experiments, and python scripts for visualizing the data.

### 1.1 Data

The Data directory contains a subdirectory for the single-story experiments and one for the two-story experiments. All data files are in comma-separated value (CSV) plain text format. The data files include time series data collected for all sensors used to instrument each structure. Data were logged at a rate of 1 Hz for the single-story experiments and 0.5 Hz for the two- story experiments. The data files appended with ‘Events’ include timestamp data from each of the ventilation events in the experiments. The ventilation events differentiated each unique experiment. Data files from replicate experiments included an ‘R’ in the file names followed by the number of the replicate experiment.

### 1.2 Information

The Information (Info) directory contains a subdirectory labeled ‘Floorplans’ with the di- mensioned floorplans of each of the structures as well as a table with the dimensions of the window and door openings. The directory also includes CSV files listing out the details about each of the sensors including the location of the sensor, data measured, and the array with which each sensor is grouped. Additional files indicate the axis limits for plotting for each measurand. This information is necessary for the plotting scripts.

### 1.3 Scripts

The Scripts directory contains python scripts for plotting the data from the single-story and two-story experiments, respectively. The data from each sensor array are plotted in a unique figure and named according to the sensor array. The scripts make a Charts directory on the same level as the Scripts directory and populate the directory with a set of subdirectories. The subdirectory structure includes a level under the Charts directory specific to the structure and a level under the structure specific to the experiment conducted in the structure. The scripts generate portable document format (PDF) files with the plots named according to the location and measurand of the sensor array.

## 2 Experimental Design, Materials and Methods

All experiments described here were conducted at full scale in purpose-built residential-style structures with variable ventilation configurations. The structures included an approximately 111 m^2^ single-story ranch structure and a 297 m^2^ two-story colonial structure. The single- story, traditional ranch-style structure was designed to represent a home constructed in the mid-twentieth century with walls and doorways separating all of the rooms and 2.44 m ceilings. The two-story, contemporary colonial-style structure was designed to represent a more modern de- sign that incorporated an open plan arrangement, a two-story foyer, a two-story family room, and four bedrooms.

All experiments featured a 0.6 m × 0.6 m natural gas burner as the fire source to provide control of the fire and hot gas plume. Each ventilation scenario consisted of opening windows and doors to the homes in a distinct sequence to generate flow paths that connected the fires with remote intake and exhaust vents. In total, three unique experiments were conducted in the single-story structure and three unique experiments were conducted in the two-story structure. Replicates of some experiments were conducted to investigate repeatability of the collected data. This work was part of a larger series of experiments that studied the impact of ventilation on fire patterns. Those data can be explored at https://fireinvestigation.fsri.org/.

### 2.1 Instrumentation

Instrumentation was installed to measure gas temperature, gas pressure, and gas velocity within the structures. Gas temperatures were measured with bare-bead Chromel-Alumel (type- K) thermocouples made from 1.3 mm diameter wire. 1.6 mm bead diameter inconel sheathed thermocouples were used in conjunction with the bi-directional probes for gas velocity mea- surements. Pressure measurements were made using differential pressure sensors to determine pressure changes relative to ambient conditions (outside of the structure). Differential pressure sensors were also used to determine gas velocity magnitude using the bi-directional probes. The differential pressure sensor was a Setra Model 264 with a range of ± 125 Pa.

All numerical data was recorded with a National Instruments data acquisition system that incorporated a SCXI-1001 chassis with eight SCXI-1102C 32-Channel modules each connected to a TC-2095 end terminal with built-in cold junction compensation for thermocouple measure- ments. The TC-2095 also accepted 0-10 V DC for non-thermocouple measurements. The system was configured for a total of 256 channels. A separate system was used for each structure. Data were logged at a rate of 1 Hz for the single-story experiments and 0.5 Hz for the two-story experiments.

### 2.2 Fire source

A single natural gas burner was the sole fire source within the unfurnished structures for all experiments. The burner had dimensions of 0.6 m by 0.6 m and the surface of the burner was located approximately 0.6 m above the floor. The burner was calibrated under a ventila- tion hood instrumented with an oxygen-depletion calorimeter. The calibration yielded a heat of combustion of approximately 45 MJ/kg for the natural gas that supplied the burner. An Alicat MCRH-5000 SLPM mass flow controller was used to ensure the mass flow rate to the burner matched the desired heat release rate. The total heat release rate for the single-story structure was 250 kW (flow rate set point of approximately 400 standard liters per minute (SLPM) with a reference temperature of 0°C), and 500 kW (flow rate set point of approximately 800 SLPM with a reference temperature of 0°C) for the two-story structure. The wall and ceiling immediately adjacent to the burner were reinforced with cement board to minimize excessive thermal damage to the structure.

### 2.3 Single-story structure

The single-story, ranch-style structure had overall interior dimensions of 13.9 m by 7.7 m. The layout of the structure included three bedrooms, a living room, a dining room, and a kitchen. The space continuous with the kitchen between the kitchen and the dining room was designated as the breakfast area. There were also areas, normally designed as a bathroom and closets, that were walled-off from the rest of the structure to provide protection to the installed instrumentation.

The walls were constructed from dimensional lumber, 38 mm by 89 mm (nominally 2 in. by 4 in.). The studs were lined with two layers of gypsum board. The base layer was 16 mm (5/8 in.) thick regular gypsum wallboard. The top layer was 13 mm (1/2 in.) thick light gypsum wallboard. The ceiling supports were constructed from engineered lumber I-joists and were covered with gypsum wallboard in the same manner as the walls. The floor was constructed from dimensional lumber, 38 mm by 89 mm (nominally 2 in. by 4 in.) and covered with a 18 mm (3/4 in.) thick plywood subfloor. On top of the plywood was 13 mm (1/2 in.) cement board. The single-story structure had two exterior doors (front and back), three bedroom doors, and a doorway that led to the kitchen. All doors and doorways had a height of approximately 2.0 m. The interior doors were hollow-core wood frame doors.

**Fig. 1:**
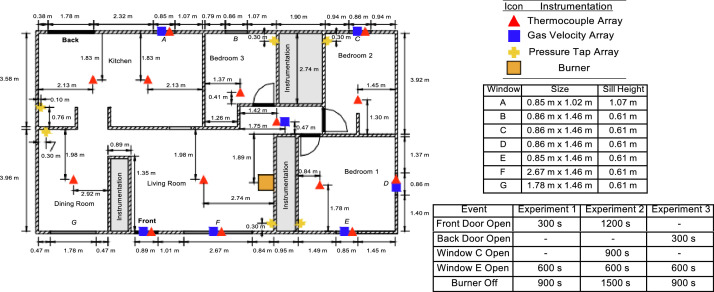
Dimensioned plan view drawing of the single-story structure indicating instrumentation locations.

The locations of the instrumentation and the natural gas burner in the ranch-style structure are displayed in Fig. 1. Each thermocouple array consisted of eight type-K thermocouples. The highest elevation thermocouple in each array was located 2.5 cm below the ceiling with the remaining seven spaced at approximately 30.5 cm intervals (30.5 cm below ceiling, 61 cm below ceiling, ..., 213 cm below ceiling). Three pressure taps were installed at each location at elevations of 30.5 cm, 122 cm, and 213 cm below ceiling.

Bi-directional velocity probes were installed in relevant doorways, windows, and the interior hallway of the single-story structure. In each case, the five probes in the array were evenly spaced along the centerline of the opening. The probes in the windows were spaced 0.24 m apart. Therefore, the probe in the window openings at the highest elevation was 0.61 m below the ceiling and the lowest elevation probe in the window openings was approximately 1.6 m below the ceiling. The probes in the front door opening were spaced 0.33 m apart. The probe in the front door opening at the highest elevation was 0.73 m below the ceiling and the lowest elevation probe was approximately 2.07 m below the ceiling. The probes in the hallway were spaced 0.4 m apart. The probe in the hallway at the highest elevation was 0.4 m below the ceiling and the lowest elevation probe was approximately 2.0 m below the ceiling. The temperature and gas velocity measurements at the front door and each of the windows were only utilized for experiments where the front door or the particular window opening of interest was vented.

### 2.4 Two-story structure

The two-story, colonial-style structure had overall interior dimensions of 15.05 m by 10.13 m. The layout of the first story included a living room, den, family room, kitchen, laundry room, dining room, closet, and entrance foyer. The first story included two areas that were walled-off from the rest of the structure that provided protection for the installed instrumentation. The second story had the same interior dimensions as the first story, with four bedrooms and three areas that were walled-off from the structure that provided protection to the installed instrumentation (these instrumentation areas included the hall bath, the bedroom 4 closet, and a combination of the master bath and closet). The areas above the first story family room and above the foyer were open to the second story.

The walls of the two-story structure were constructed from dimensional lumber, 38 mm by 89 mm (nominally 2 in. by 4 in.). The studs were lined with two layers of gypsum board. The base layer was 16 mm (5/8 in.) thick regular gypsum wallboard. The top layer was 13 mm (1/2 in.) thick lightweight gypsum wallboard. The ceiling supports for the upper story were constructed from engineered lumber I-joists and were covered with gypsum wallboard in the same manner as the walls. The floor for the upper story was supported by dimensional joists, 38 mm by 286 mm (nominally 2 in. by 12 in.) and covered with 18 mm (3/4 in.) thick plywood subfloor. The floor of the lower level was constructed from dimensional lumber, 38 mm by 89 mm (nominally 2 in. by 4 in.) and covered with 18 mm (3/4 in.) thick plywood sub floor. On top of the plywood was 13 mm (1/2 in.) cement board.

**Fig. 2:**
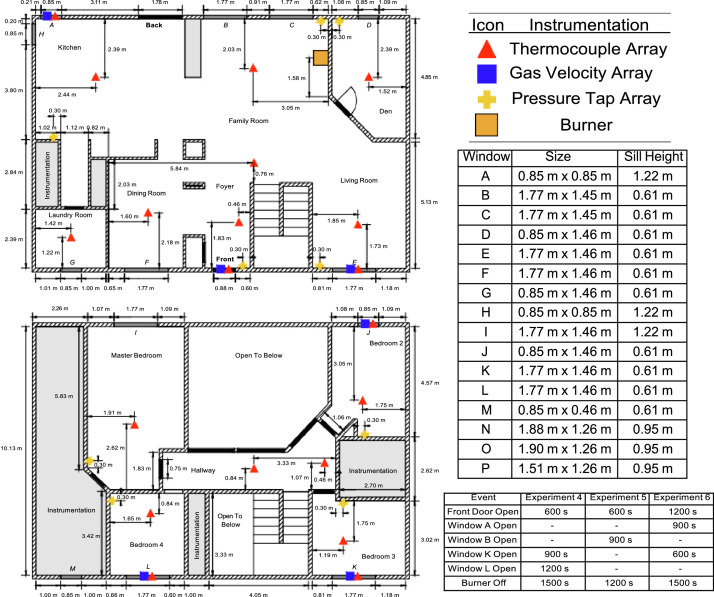
Dimensioned plan view drawing of the two-story structure indicating instrumentation locations.

The locations of the instrumentation and the natural gas burner in the colonial-style structure are displayed in Fig. 2. The two-story structure had two exterior doors (front and back) and six interior doors (four bedrooms, laundry room, and den). All doors had a height of approximately 2.0 m. The interior doors were hollow-core wood frame doors. All interior doors were open for all experiments, with the exception of the door to the den, which was closed in Experiment 6.

Instrumentation in the two-story structure remained constant throughout all the experiments. Thermocouple arrays consisted of either eight or 16 type-K thermocouples. There were two 16-thermocouple arrays installed in the two-story structure: one in the family room and one in the foyer. The remainder of the thermocouple arrays had eight sensors. In all cases, the highest elevation thermocouple in each array was located 2.5 cm below the ceiling with the remaining thermocouples spaced at 30.5 cm intervals. The lowest elevation thermocouples in the eight- thermocouple arrays were approximately 213 cm below the ceiling and the lowest elevation thermocouples in the 16-thermocouple arrays were approximately 460 cm below the ceiling. Three pressure taps were installed at each location at elevations of 30.5 cm, 122 cm, and 213 cm below the ceiling in all locations except the family room. Because the family room was open to the second story, the three elevations at which pressures were measured were 30.5 cm, 244 cm, and 460 cm below the ceiling. Bi-directional velocity probes were installed in relevant doorways and windows. In each case, the five probes in the array were evenly spaced within the respective location.

## CRediT authorship contribution statement

**Mark McKinnon:** Writing – original draft, Visualization. **Craig Weinschenk:** Data curation, Investigation. **Daniel Madrzykowski:** Writing – review & editing, Supervision. **Keith Stakes:** Investigation, Methodology.

## Declaration of Competing Interest

The authors declare that they have no known competing financial interests or personal relationships which have, or could be perceived to have, influenced the work reported in this article.

## Appendix Supplementary materials

Image, application 1
