# Supplementary material for: Gas burner experiments conducted in modern residential style structures
Source: Data Brief. 2021 Nov 23;39:107624. doi: 10.1016/j.dib.2021.107624 (PMC8637480; doi:10.1016/j.dib.2021.107624)
Supplement: Supplementary file 1 [file mmc1.zip › DiB_colonial_alt.pdf]

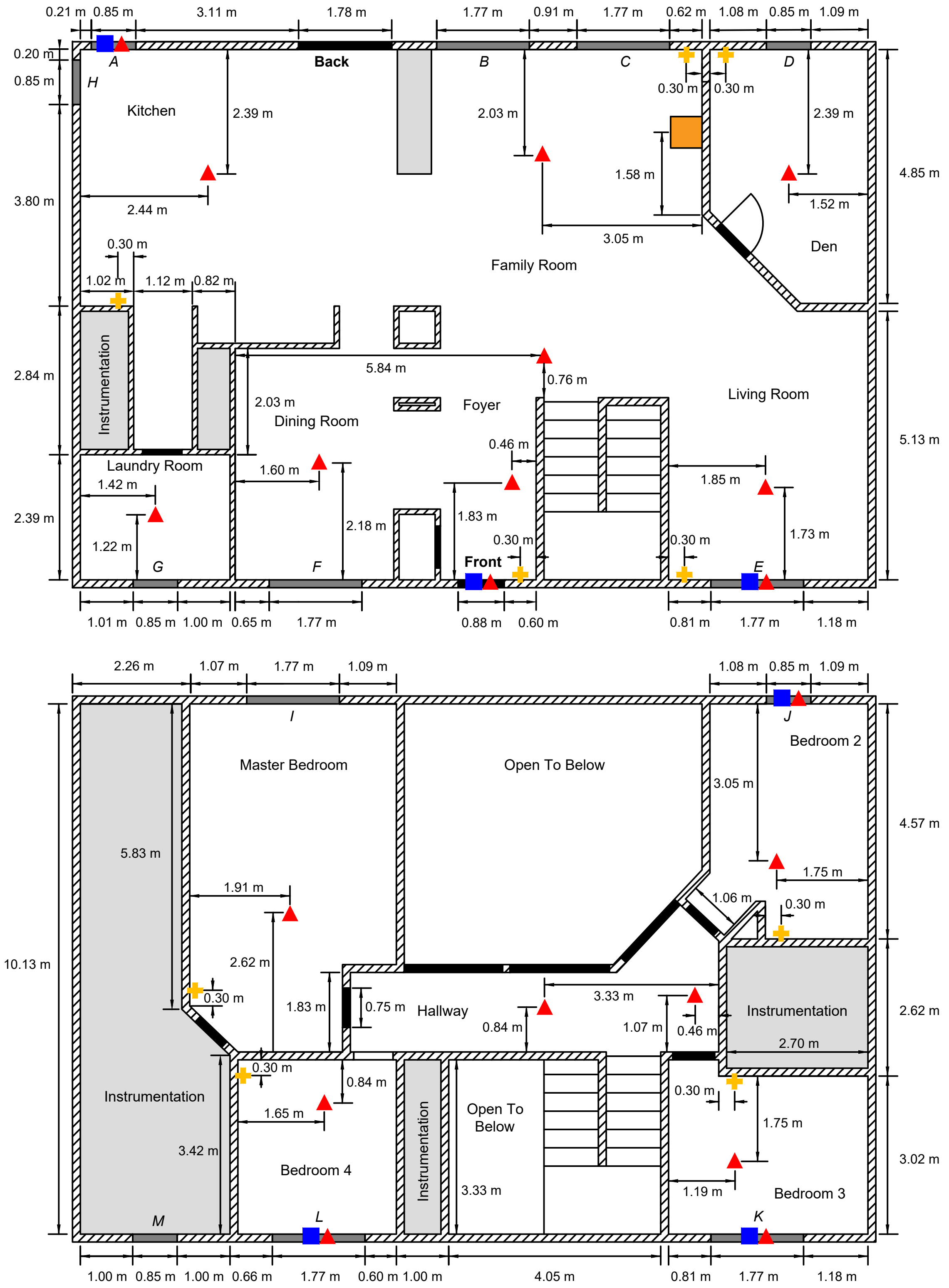

Icon	Instrumentation
	Thermocouple Array
	Gas Velocity Array
	Pressure Tap Array
	Burner

Window	Size	Sill Height
A	0.85 m x 0.85 m	1.22 m
B	1.77 m x 1.45 m	0.61 m
C	1.77 m x 1.45 m	0.61 m
D	0.85 m x 1.46 m	0.61 m
E	1.77 m x 1.46 m	0.61 m
F	1.77 m x 1.46 m	0.61 m
G	0.85 m x 1.46 m	0.61 m
H	0.85 m x 0.85 m	1.22 m
I	1.77 m x 1.46 m	1.22 m
J	0.85 m x 1.46 m	0.61 m
K	1.77 m x 1.46 m	0.61 m
L	1.77 m x 1.46 m	0.61 m
M	0.85 m x 0.46 m	0.61 m
N	1.88 m x 1.26 m	0.95 m
O	1.90 m x 1.26 m	0.95 m
P	1.51 m x 1.26 m	0.95 m

Event	Experiment 4	Experiment 5	Experiment 6
Front Door Open	600 s	600 s	1200 s
Window A Open	-	-	900 s
Window B Open	-	900 s	-
Window K Open	900 s	-	600 s
Window L Open	1200 s	-	-
Burner Off	1500 s	1200 s	1500 s
