# Supplementary figures and images for: Gas burner experiments conducted in modern residential style structures
Source: Data Brief. 2021 Nov 23;39:107624. doi: 10.1016/j.dib.2021.107624 (PMC8637480; doi:10.1016/j.dib.2021.107624)

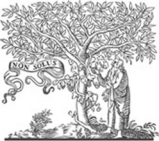

ELSEVIER

Supplement: Supplementary file 1 [file mmc1.zip › elsevier-logo.pdf]
