# Supplementary material for: Gas burner experiments conducted in modern residential style structures
Source: Data Brief. 2021 Nov 23;39:107624. doi: 10.1016/j.dib.2021.107624 (PMC8637480; doi:10.1016/j.dib.2021.107624)
Supplement: Supplementary file 1 [file DiB_ranch_alt.pdf › DiB_ranch_alt.pdf]

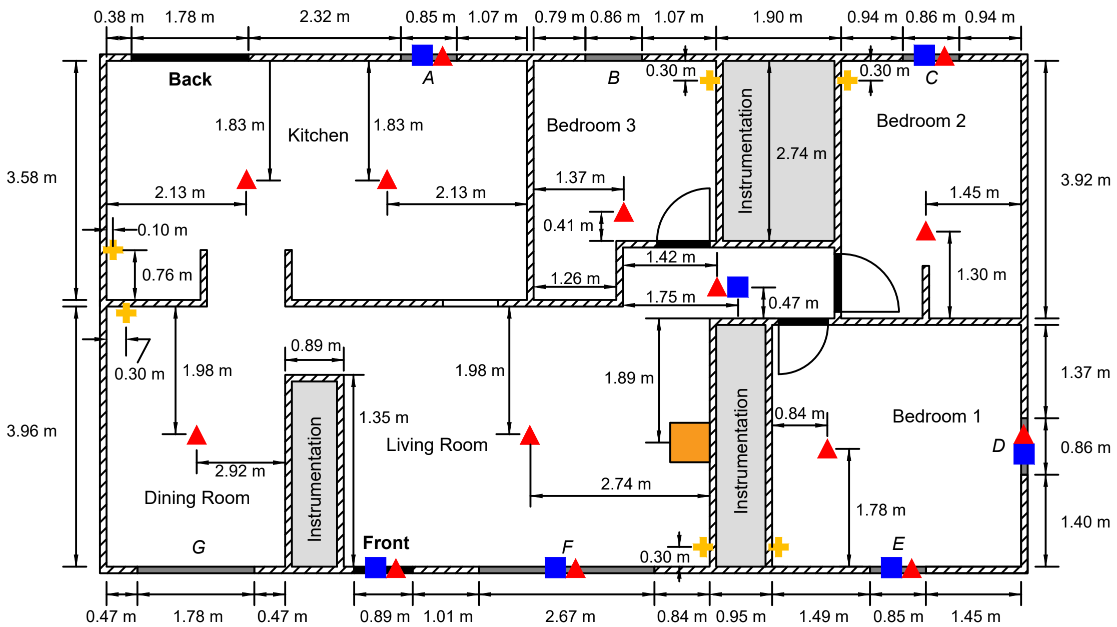

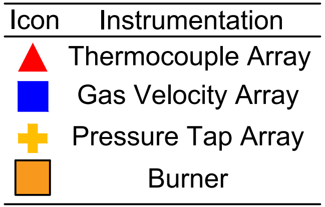

Window	Size	Sill Height
A	0.85 m x 1.02 m	1.07 m
B	0.86 m x 1.46 m	0.61 m
C	0.86 m x 1.46 m	0.61 m
D	0.86 m x 1.46 m	0.61 m
E	0.85 m x 1.46 m	0.61 m
F	2.67 m x 1.46 m	0.61 m
G	1.78 m x 1.46 m	0.61 m

Event	Experiment 1	Experiment 2	Experiment 3
Front Door Open	300 s	1200 s	-
Back Door Open	-	-	300 s
Window C Open	-	900 s	-
Window E Open	600 s	600 s	600 s
Burner Off	900 s	1500 s	900 s
